# Cortical Alpha Oscillations Predict Speech Intelligibility

**DOI:** 10.3389/fnhum.2017.00088

**Published:** 2017-02-24

**Authors:** Andrew Dimitrijevic, Michael L. Smith, Darren S. Kadis, David R. Moore

**Affiliations:** ^1^Otolaryngology—Head and Neck Surgery, Sunnybrook Health Sciences CentreToronto, ON, Canada; ^2^Hurvitz Brain Sciences, Evaluative Clinical Sciences, Sunnybrook Research InstituteToronto, ON, Canada; ^3^Faculty of Medicine, Otolaryngology—Head and Neck SurgeryUniversity of Toronto, Toronto, ON, Canada; ^4^Communication Sciences Research Center, Cincinnati Children’s Hospital Medical CenterCincinnati, OH, USA; ^5^Speech and Hearing Sciences, University of WashingtonSeattle, WA, USA; ^6^Pediatric Neuroimaging Research Consortium, Cincinnati Children’s Hospital Medical CenterCincinnati, OH, USA; ^7^Division of Neurology, Cincinnati Children’s Hospital Medical CenterCincinnati, OH, USA; ^8^Department of Pediatrics, University of Cincinnati, College of MedicineCincinnati, OH, USA; ^9^Department of Otolaryngology, University of CincinnatiCincinnati, OH, USA

**Keywords:** speech in noise, EEG, digits in noise, hearing, brain, attention

## Abstract

Understanding speech in noise (SiN) is a complex task involving sensory encoding and cognitive resources including working memory and attention. Previous work has shown that brain oscillations, particularly alpha rhythms (8–12 Hz) play important roles in sensory processes involving working memory and attention. However, no previous study has examined brain oscillations during performance of a continuous speech perception test. The aim of this study was to measure cortical alpha during attentive listening in a commonly used SiN task (digits-in-noise, DiN) to better understand the neural processes associated with “top-down” cognitive processing in adverse listening environments. We recruited 14 normal hearing (NH) young adults. DiN speech reception threshold (SRT) was measured in an initial behavioral experiment. EEG activity was then collected: (i) while performing the DiN near SRT; and (ii) while attending to a silent, close-caption video during presentation of identical digit stimuli that the participant was instructed to ignore. Three main results were obtained: (1) during attentive (“active”) listening to the DiN, a number of distinct neural oscillations were observed (mainly alpha with some beta; 15–30 Hz). No oscillations were observed during attention to the video (“passive” listening); (2) overall, alpha event-related synchronization (ERS) of central/parietal sources were observed during active listening when data were grand averaged across all participants. In some participants, a smaller magnitude alpha event-related desynchronization (ERD), originating in temporal regions, was observed; and (3) when individual EEG trials were sorted according to correct and incorrect digit identification, the temporal alpha ERD was consistently greater on correctly identified trials. No such consistency was observed with the central/parietal alpha ERS. These data demonstrate that changes in alpha activity are specific to listening conditions. To our knowledge, this is the first report that shows almost no brain oscillatory changes during a passive task compared to an active task in any sensory modality. Temporal alpha ERD was related to correct digit identification.

## Introduction

Hearing in humans is normally quantified using pure tone audiometry, which measures absolute sensitivity across a wide range of pure tone frequencies centered on those thought most useful for speech perception (Moore, 2013). However, the resulting audiogram does not provide a complete picture of listening abilities encountered in everyday environments. For example, a person with a normal pure tone audiogram may still experience difficulty understanding speech in a noisy and reverberant room (Ruggles and Shinn-Cunningham, 2011). Listening to speech in noise (SiN) is a challenging and complex task involving a high level of cognitive as well as sensory processing in the ear, the central auditory system, and multimodal areas in the temporal, frontal and parietal cortex (Hickok and Poeppel, 2007; Füllgrabe et al., 2015; Evans et al., 2016). Understanding the neural processes associated with listening to SiN has the potential to provide new clinical measures for the existence of “hidden hearing loss”, a blanket term for hearing problems not predicted by the audiogram (Kujawa and Liberman, 2009; Schaette and McAlpine, 2011; Mehraei et al., 2016). Such understanding may also help dissociate mechanisms in populations who have similar behavioral performance on SiN tests, but differ in the underlying source of dysfunction. For example, a patient with auditory neuropathy (Starr et al., 1996) arising from inner hair cell or auditory nerve dysfunction may be indistinguishable on a SiN test from a patient who has “auditory processing disorder” (APD; Moore and Hunter, 2013; Moore, 2015) arising from dysfunction at higher levels of the nervous system.

Difficulty with SiN despite normal audiograms has been estimated to occur in 5%–10% of adults who seek audiological services (Kumar et al., 2007; Hind et al., 2011). In addition, many older adults who do not necessarily seek audiological evaluation have disproportionately poor speech perception and auditory temporal processing skills than younger listeners, relative to their audiometric hearing level (Füllgrabe et al., 2015). Variations in SiN ability are also seen in young, college-aged normal hearing (NH) adults (Kidd et al., 2007). The SiN variability in this population did not appear to be related to academic ability or to spectral or temporal auditory processing skills. Some of this variation has been attributed to individual differences in phase locking ability in response to amplitude modulated tones (Ruggles et al., 2011) or frequency following response synchrony to speech fundamental frequency (F_0_) in short CV syllables (Anderson et al., 2013) of auditory brainstem neurons.

The purpose of the study reported here was to examine cortical electrophysiological processes associated with a commonly used test of SiN hearing, the digits-in-noise (DiN) test (Smits and Houtgast, 2005; Smits et al., 2013). We focused on the use of cortical oscillatory potentials to assess cognitive processing associated with SiN performance. Recent work has suggested that scalp-recorded alpha rhythms in the 8–12 Hz range are associated with a number of cognitive processes including visual attention (Thut et al., 2006), working memory (Bonnefond and Jensen, 2012), and attentive listening (Weisz et al., 2011). Alpha rhythms may be a mechanism by which the brain directs information processing (Jensen and Mazaheri, 2010). According to this hypothesis, low alpha power represents a state of activation whereas high alpha power represents a state of inhibition, gating information flow from one region of cortex to another.

A large literature exists on alpha rhythms for sensory processing in the visual system (Lleras et al., 2011). In the auditory modality, by contrast, there have been many fewer studies. Nonetheless, some key replicable findings have emerged. In an early MEG study, Lehtelä et al. (1997) observed that ongoing activity of 6.5–9.5 Hz originating from the temporal lobe was suppressed when listeners were presented with a sound. Similar findings were reported in children passively listening to noise bursts and violin sounds (Fujioka and Ross, 2008). Decreases in alpha relative to baseline, event-related desynchronization (ERD), started 500 ms after stimulus onset and lasted 750 ms. Inferred sources of this ERD were consistent with auditory cortex. In summary, these findings suggest that temporal alpha is related to auditory processing in quiet or in noise.

More recently, alpha power modulation has been explored under various cognitively demanding tasks requiring memory retention or spatial attention, for example listening to degraded or masked speech. These demanding tasks are thought to result in increased “listening effort” (Pichora-Fuller et al., 2016). Obleser and Weisz (2012) found a prominent alpha ERD lasting up to 1 s while listening to spectrally degraded speech sounds. Greater ERD was associated with better comprehension. During SiN, a consistent finding of increased alpha power relative to baseline (event-related synchronization, ERS) has been found (Wilsch et al., 2015; McMahon et al., 2016). In the McMahon et al. (2016) study, alpha ERS was associated with increased pupil dilation, thought to be associated with increased listening effort (Zekveld et al., 2010). One interpretation of these findings has been that ERS occurs as a result of suppressing noise encoding “channels” in the auditory system thereby “protecting” the auditory input to be attended (Strauß et al., 2014). Changes in alpha were also associated with effortful listening in degraded conditions that draws upon shared cognitive resources including working memory and attention.

The DiN presents successive trials of three digits mixed with an unmodulated speech-shaped noise. Signal to noise ratio (SNR) is varied adaptively. We chose to study the DiN because: (i) it is very widely used, with over two million tests taken in at least eight languages worldwide, and four dialects of English (personal communication, Dr. De Wet Swanepoel, University of Pretoria); (ii) it is internet and smartphone deliverable and has been used in several large scale studies (Smits and Houtgast, 2005; Moore et al., 2014; Louw et al., 2016); (iii) it is cognitively and linguistically undemanding, and offers the possibility of obtaining comparable data across language groups (Smits et al., 2016) and a wide range of ages (4–90 years; personal communication, Dr. Cas Smits, Free University of Amsterdam); and (iv) it consists of temporal and detection threshold balanced stimuli that are well suited for EEG research.

This report is the first of which we are aware that documents continuous EEG during administration of a SiN test. The aim is to provide and interpret cortical records of speech hearing in noise with a long term goal of developing a scientifically and clinically useful tool to dissect the sensory and cognitive neural contributions to listening to a realistic auditory task.

## Materials and Methods

### Participants

Fourteen adult participants (10 females; mean age: 25.4 years) were recruited through Cincinnati Children’s Hospital Medical Center, according to an Institutional Review Board (IRB) approved protocol. Participants were screened for audiometric hearing thresholds ≤20 dB HL bilaterally at octave test frequencies from 250 Hz to 8000 Hz, and had no clinically significant neurological or mental health issues. Participants received a monetary incentive and provided informed consent in accordance with the Declaration of Helsinki.

### Stimuli and Procedure

#### Digits

Procedures and rationale for recording, equalizing, and homogenizing the speech and noise stimuli have been presented in detail previously (e.g., Smits et al., 2004; Vlaming et al., 2014). All speech stimuli were recorded from a female talker of standard American English using Computerized Speech Lab (CSL) hardware and software (Kay Elemetrics, 2001). The speech stimuli consisted of an introductory phrase “The numbers” and monosyllabic digits 0–9 excluding the disyllabic 7, where the “0” was pronounced “Oh” (/oω/). In order to preserve natural prosody, the digits were recorded in series of triplets. The talker read lists of digit triplets with the introductory phrase. The list was organized such that each digit occurred in each of the three positions (i.e., as the first, second or third read digit). In Adobe Audition, each digit in each position was extracted and a .wav file was created. These recordings were reviewed and exemplar digits were chosen for each position. The criteria for choosing the exemplar digits included a subjective determination of quality; lack of prosodic irregularities, hesitations and lack of any acoustic distortion. Measured durations varied from 434 ms to 672 ms (SD 57 ms). Silence was introduced at the end of each digit such that the overall duration of the digit file was the same (695 ms). The process created 27 unique digit files (nine digits, for each of the three positions). The amplitude of the sound component of each file was adjusted by a scale factor to equalize rms across all digits. The long-term average speech spectrum for all nine digits was mixed to create spectrally matched noise maskers.

**Figure 1:**
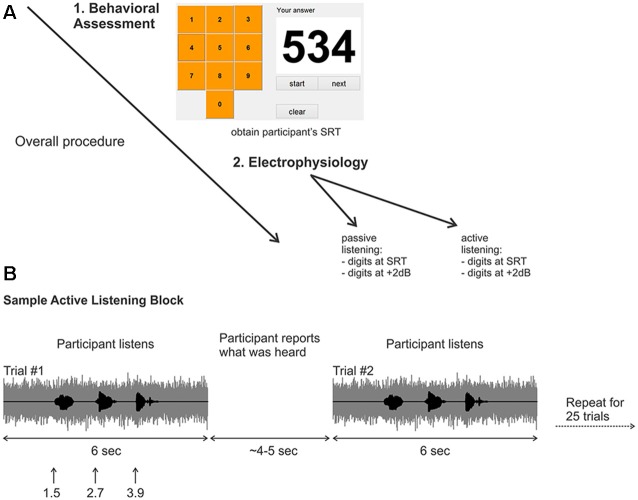
**(A)** Overall experimental procedure. First, a behavioral assessment was performed on each participant to obtain the digits-in-noise (DiN) speech reception threshold (SRT). Afterwards, the electrophysiology was performed using the subject-specific SRT or +2 dB. **(B)** Two sample trials in the active listening portion of the experiment. The signal to noise ratio (SNR) was kept constant at the SRT or +2 dB. After the DiN was presented, the participant verbally reported what digits were perceived. The experimenter recorded the participant’s response then initiated the next trial. This was repeated for a total of 25 trials in each run. Eight runs were recorded in total resulting in 200 trials per condition, (for SRT, ~100 correct and ~100 correct).

A final step was a stimulus homogenization procedure (Smits et al., 2004; Vlaming et al., 2014) that equated the audibility of the digits in noise. These steps were necessary because the speech reception threshold (SRT) measure used in the DiN is based on equal audibility of all the digits. Homogenization was accomplished by creating psychometric functions, the percent correctly identified digits as a function of SNR for each of the digits presented separately and pseudo-randomly, using a constant noise masker level (75 dBA) and varying the sound level of the digit. All stimuli were calibrated in a 2 cc coupler with a Brüel and Kjær (model 2260) sound level meter set on slow time weighting. The SNRs used for this procedure were stepped from −20 dB (near 10% digits identified) to 0 dB (near 100% digits identified). In a 10-alternative (digits 0–9, see Figure 1) identification paradigm, listeners were asked to provide a response, even if they guessed. The psychometric functions were modeled using a sigmoid function and the SNR for 50% identification level was computed. This procedure was completed for 10 NH adults (mean age 25). The computed mean SNR amplitude for 50% performance was then used as a final scaling factor for each digit.

#### Digits in Noise Test (DiN)

A customized Matlab program was designed to present the triplet digits in noise in successive trials, enabling the estimation of SRT, defined as the SNR yielding 50% correct identification for each set of three digits (Smits et al., 2004) A graphical user interface (GUI) resembling a telephone touch key pad (i.e., 3 rows and 3 columns for digits 1–9 and bottom middle for the 0 digit; see Figure 1A) was incorporated for user response after stimulus presentation. The user initiated the beginning of the test and heard the noise masker, then the carrier phrase “The numbers”. The first digit occurred 1 s later, followed by the second and third digits (ISI = 500 ms; (Figure 1B). The noise masker was turned off 1 s after the offset of the third digit. The entire stimulus (noise and masker) lasted 6 s. The GUI then allowed the participant to indicate which digits were heard. The SNR of successive triplets was varied adaptively from an initial level of +2 dB. Trials following a correct response (all three digits) reduced the SNR by 2 dB (noise constant, digit amplitudes reduced). Incorrect responses were followed by an increased SNR, also by 2 dB. Twenty-five trials were presented and the average SNR over the last 11 trials was the SRT. All sounds were presented diotically through ER3 insert earphones with a fixed speech masker level of 75 dBA.

#### EEG

Electrophysiological recordings were performed after the behavioral SRT determination. EEG recordings used the same stimuli as behavioral testing, except that no introductory phrase was used and all trials had the same SNR. In the attentive listening task, rather than responding with the GUI, the participant verbally reported heard digits. The experimenter then initiated the next trial. Two levels of SNR were used, +2 dB (performance at 100% for all three digits) and previously measured SRT (performance close to 50% for all three digits, see below). Only data using the SRT level are presented here. Our pilot behavioral data suggested that participants perform better (lower SRTs) with repeated testing (see Smits et al., 2013; Vlaming et al., 2014). If we had used the SRT estimated from the behavioral test (25 trials), in our EEG testing (200 trials) most participants would identify the three digits with ~75%–90% accuracy. Because we aimed to have roughly an equal number of correct and incorrectly identified trials (100 each) it was problematic to determine *a priori* what SNR should be used. Based on pilot data, we adopted a threshold seeking approach using two blocks of 25 trials (50 trials), with a starting SNR 2 dB below the behavioral SRT. If in these two blocks, the percent correct was in the 40%–60% range, we continued with this SNR (8/14 participants). In the remaining six participants, the EEG SNR was 3 dB less than the behavioral SRT (i.e., more negative).

The overall procedure is summarized in Figure 1. Recordings were performed in eight blocks of 25 trials yielding 200 trials for each randomly chosen stimulus condition (i.e., 200 trials for the SRT and 200 trials for +2 dB). Two listening conditions were assessed; “passive” listening, where the participants were instructed to ignore any sounds while they watched a closed caption and silent movie of their choice, and an attentive (“active”) listening condition where participants fixated a white cross on a computer screen and repeated verbally all the digits presented (Figure 1B). During the active listening condition, the three perceived digits for each trial were recorded by the experimenter, who then initiated the next trial. The active listening condition (eight blocks per SNR) always occurred first, followed by the passive listening condition (eight blocks per SNR). Participants were encouraged to take breaks after each block.

The electrophysiological data were collected using a 64-channel actiCHamp Brain Products recording system (Brain Products GmbH, Inc., Munich, Germany). An electrode cap was placed on the scalp with electrodes placed at equidistant locations (Han and Dimitrijevic, 2015). The infracerebral cap used covers a larger area than is typical in a 10–20 system. The reference channel was located at vertex (Cz) while the ground electrode was located on the midline 50% of the distance to nasion. Continuous data were digitized at 1000 Hz and stored for offline analysis.

### Data Processing

#### Preprocessing

The electrophysiological data were first processed using Brain Vision Analyzer ver. 2.0 (Brain Products GmbH, Inc., Munich, Germany). Data were high-pass filtered (0.1 Hz) to remove baseline drifts and down sampled to 250 Hz. Visual inspection and manual sorting of the data included removal of extreme stereotypical artifacts related to subject movement (exceeding 500 mV). Independent component analysis (ICA; Delorme and Makeig, 2004), as implemented in Brain Vision Analyzer (identical algorithm to EEGLAB; Delorme and Makeig, 2004), was applied to reduce ocular and cardiac artifacts. On average four independent components were removed per subject.

#### Time-Frequency Analysis

Data were averaged, referenced and segmented into epochs −1500 ms to 7000 ms relative to speech masker onset. All time-frequency analyses were performed in BESA 6.0 (Brain Electrical Source Analausi, GmbH, Germany) using a 50 ms window with a 2 Hz frequency resolution.

#### Brain Source Analysis

After the time-frequency analysis, a beamformer, as implemented in BESA was applied to a time-frequency region of interest. The choice of the time-frequency region of interest was based on the condition specific grand mean time-frequency analysis.

#### Statistical Analysis

All statistical analyses were performed in BESA Statistics 2.0 in a similar manner to that previously described (Han and Dimitrijevic, 2015). Differences between conditions (beamformer source) were assessed by performing a paired *t*-test in source space and then corrected for multiple comparisons using Monte-Carlo resampling techniques (Maris and Oostenveld, 2007). Clusters of voxels with *p*-values of less than 0.05 were considered significant.

## Results

### Effects of Attention

**Figure 2:**
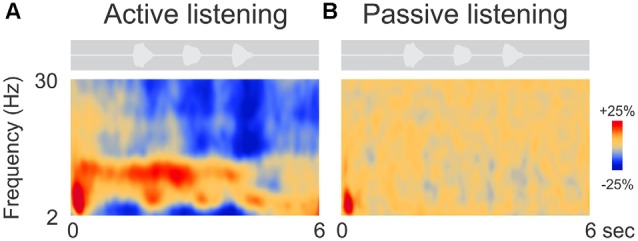
**Grand mean time-frequency representations of the electrophysiological data.** The left **(A)** side shows the mean trial data from the active listening condition whereas, on the right **(B)**, data from the passive listening are shown. The DiN stimulus is shown above each plot indicating the timing of the stimuli relative to the time-frequency plot. Time-frequency plots have a number of different illustration conventions. In this case, oscillatory changes are calculated as a percent change from baseline. Oscillatory activity is shown as increased (red; event-related synchronization, ERS) or decreased (blue; event-related desynchronization, ERD) activity relative to baseline (not shown). Note that the active condition is characterized by more oscillatory power in the alpha (8–12 Hz), beta (15–30 Hz) delta/theta (2–6 Hz) bands than in the passive listening condition. Time frequency data were averaged across all 63 electrodes.

Figure 2 shows the grand mean time-frequency representations averaged across 63 electrodes and all 14 participants. After an initial onset response to the noise in both conditions (red, low frequency deflection), oscillations during digit presentation were apparent only during the attend condition and predominantly in the alpha (8–12 Hz) frequency domain. Other frequency changes, including beta (15–30 Hz) and lower frequency (2–6 Hz, near delta and theta) ERD (Figure 2A) were also observed. These effects will not be discussed further.

### Individual Variability

**Figure 3:**
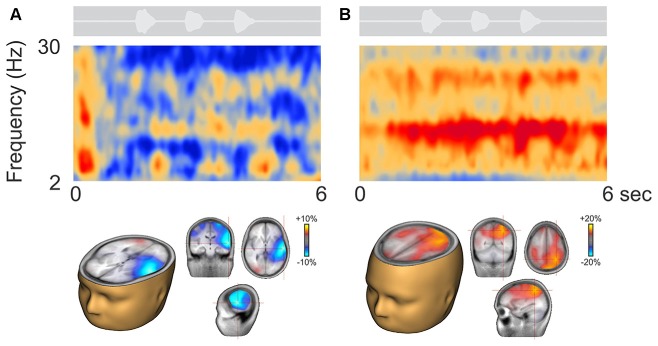
**Representative participants during active listening.** Variability in the time-frequency representations were observed. Some participants, had alpha ERD that localized to temporal/auditory regions **(A)** while others had more ERS that localized to central or parietal regions **(B).** On average, the ERD was of smaller magnitude than the ERS (note the scale differences; see also Figure 4A).

Figure 3 shows two representative participants during active listening, one (Figure 3A) showing predominant ERD and the other (Figure 3B) ERS. In some cases, ERD during digit presentation was seen across a wide range of oscillations (2–30 Hz; Figure 3A). Alpha ERD in this case was largely restricted to the left temporal cortex, with a small patch of ERS in the right occipital region. For the ERS case (Figure 3B) a second band of oscillation near the first harmonic (~25 Hz) of the alpha ERS was observed. Alpha ERS was more broadly distributed across the brain, with activity focused in the left posterior parietal and occipital cortex, and the right parietal cortex.

**Figure 4:**
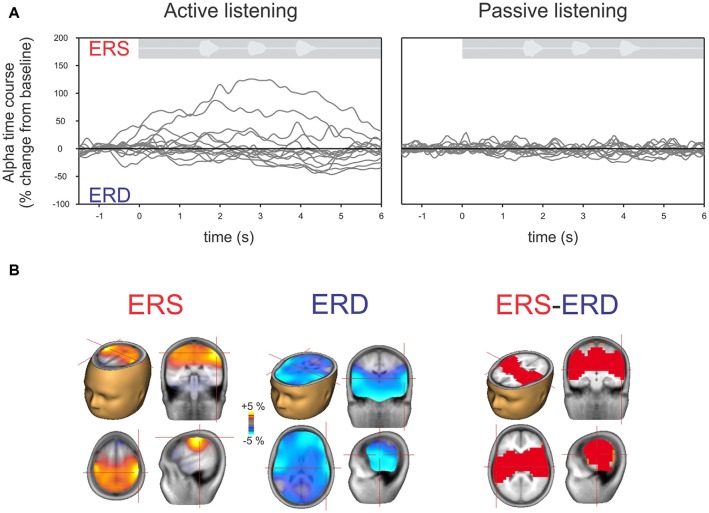
**(A)** Time course of alpha (8–12 Hz) across individual listeners shown separately for active (left) and passive (right) listening. Each gray line represents a single subject’s 63-channel averaged alpha activity. By definition, increases above the baseline are defined as ERS and below the baseline as ERD. Note that, similar to Figure 2, hardly any deviations from the baseline are seen with passive listening compared to active listening where some show ERS and others ERD. **(B)** Mean source activation of alpha (8–12 Hz, over a 2–3 s window) is shown for those who had ERS and those who had ERD. The source of ERS is predominantly in the central/parietal regions whereas the ERD is predominately in the temporal/auditory regions. A statistical comparison between those with ERS vs. ERD showed that differences are mostly seen in the auditory/temporal regions vs. central/parietal regions (*p* < 0.05).

Figure 4A shows the time course of alpha changes (mean of 63 electrodes) relative to baseline during active (left) and passive (right) listening with all individuals overlaid. Because the ERS is of larger magnitude, the grand mean data shows an overall ERS (Figure 2). Note, however, that just two individuals (of 14 tested) produced the high amplitude ERS, with a time course corresponding to the period of sound delivery. Figure 4B shows the brain source reconstruction of those participants who showed a predominant alpha ERS (*n* = 5) or alpha ERD (*n* = 9) during active listening. The alpha ERS was predominantly localized to central/parietal regions whereas the ERD was localized to the temporal and inferior frontal regions. An unpaired *t*-test showed that the differences between the ERS and ERD generators were predominantly bilateral in the temporal lobes, suggesting that the medial/temporal ERS bilateral sources were variable among participants.

### Alpha Power and Digit Identification

**Figure 5:**
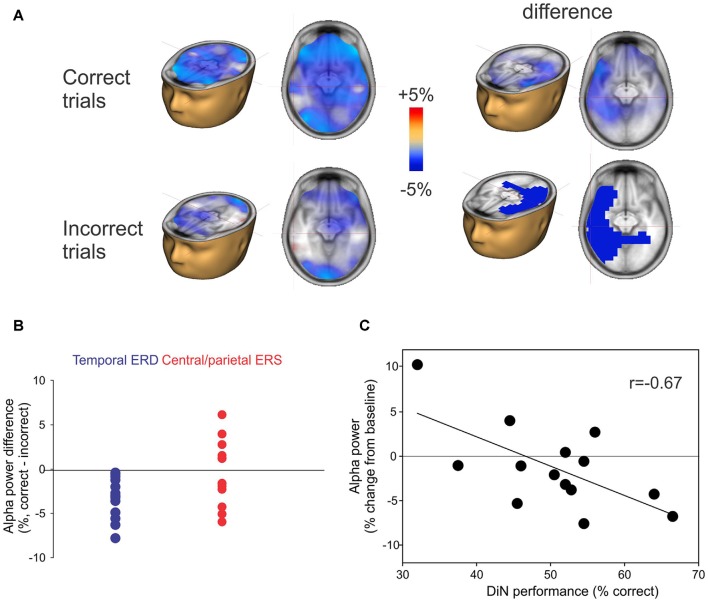
**(A)** Correct or incorrect identification of digits. Active listening trials were sorted and separately averaged depending on identification of all three digits. Significant differences in alpha sources were observed between correct and incorrect DiN trials. On the left, the alpha ERD power was greater in magnitude on the correct trials vs. incorrect trials. The difference (correct minus incorrect trials) are shown on the top right and significant (*p* < 0.05) clusters are shown below. **(B)** Individual peak difference in temporal (blue) alpha (correct minus incorrect) and central/parietal (red) alpha. The central/parietal alpha showed no consistent difference (correct vs. incorrect) whereas the temporal alpha was always greater in magnitude (more negative) on the correct than on incorrect trials. **(C)** A significant correlation was found between the alpha power (averaged across all trials, correct and incorrect) and DiN performance. The alpha power was computed at Talairach coordinates (−32, 4, 24), this location was found to have the peak correlation with DiN performance and alpha.

The alpha ERD was of greater magnitude in left temporal regions when the participant correctly identified the digits (Figure 5A; top row) whereas the alpha ERS (also 8–12 Hz, 2–3 s) showed no consistent difference when the digits were identified correctly. No significant correlations between DiN performance (correct/incorrect) and alpha power were observed at individual scalp electrodes. Figure 5B shows the difference in alpha power between correct and incorrect trials for all participants. The temporal ERD was consistently more negative (greater magnitude) for the correct trials. On the other hand, the central/parietal alpha ERS showed no consistent differentiation between correct and incorrect digit identification. Figure 5C shows a significant negative correlation between alpha ERD and DiN performance. The EEG data were obtained by performing a time-frequency analysis on all trials (correct and incorrect) for all participants, then determining the source and voxel strength of alpha (8–12 Hz, 2–3 s). The Talairach location (−32, 4, 24) indexed the maximum correlation and this value was used to extract alpha power (percent change from baseline) for each participant. These data show that greater alpha ERD is associated with superior DiN performance.

## Discussion

This study sought to characterize EEG frequency-domain activity while performing a SiN task. Three novel findings were: (1) attentive listening produced robust alpha oscillatory ERS activity in some listeners, whereas passive listening produced almost no induced oscillatory activity in any listener; (2) there was considerable variability between listeners during active listening, with ERD dominant in some listeners. The magnitude of ERD was lower than that of ERS resulting in an overall ERS in the grand mean; and (3) DiN performance was related to alpha ERD in temporal lobe but not to ERS observed in central/parietal regions.

### Effects of Attention Alpha ERS and ERD

In both passive and active listening conditions, participants were presented with three random digits in noise stimuli and only the focus of attention changed (from the closed captioned movie to the digits). The emergence of these oscillations only when attention was focused to the digits during constant sensory stimulation suggests that the oscillations arise from neural processes associated with selective attention and not with the physical characteristics of the sounds. To our knowledge this effect has not been previously reported in any sensory modality. Typically, selective attention paradigms have compared attention differences when a particular focus of attention is altered (e.g., left ear vs. right ear) yielding the robust and classic N1 enhancement for the attended vs. unattended side (Hillyard et al., 1973). In recent years, the alpha rhythm has been hypothesized to be a mechanism by which the brain alters neural activation from task-irrelevant regions through ERS and ERD (Jensen and Mazaheri, 2010). An increase in alpha (ERS) is thought to represent inhibition whereas a decrease in alpha (ERD) is thought to represent neural activation (Pfurtscheller and Lopes da Silva, 1999). In parallel to selective attention to ear of stimulation, others have reported changes in alpha activity related to left- vs. right-sided selective attention (Kerlin et al., 2010; Weisz et al., 2013; Wöstmann et al., 2016). Overall, these studies show a relation of alpha power to selective spatial attention where increases in alpha represent neural suppression contralateral to the ignored stimuli and a decrease in alpha in the hemisphere contralateral to the “to be attended” stimuli (reviewed in Strauß et al., 2014). Our data suggest that alpha oscillatory changes are also a marker for selective and sustained object attention during SiN.

### Source Localization

We observed alpha ERS originating from central/parietal regions and alpha ERD originating from temporal regions adjacent to and overlapping with auditory cortex. These cortical sources are consistent with previously described sources for speech-sound evoked alpha ERS and ERD. For alpha ERD, Becker et al. (2013) found left temporal alpha suppression (ERD) during listening to degraded (vocoded) speech. Similar, temporal alpha ERD sources were found in a study in which participants rated the comprehensibility of degraded words (Obleser and Weisz, 2012). This study also found more distributed frontal and parietal sources. We interpret the increased alpha ERD to represent a top down control of increased sensory gain (Fritz et al., 2007), analogous to the classic “N1 effect” showing an increase in amplitude with selective attention (Hillyard et al., 1973). This interpretation is also consistent with fMRI studies indicating increased activation of primary cortical regions when comparing attentive and passive listening conditions (Grady et al., 1997; Jäncke et al., 1999).

The alpha ERS we observed is qualitatively similar to previously reported alpha ERS in a variety of experimental masking paradigms (Obleser et al., 2012; Petersen et al., 2015; Wilsch et al., 2015; Wöstmann et al., 2015; McMahon et al., 2016). Not all of these studies presented or inferred sources of the alpha ERS. However, McMahon and colleagues observed a prominent alpha ERS over parietal electrodes during a sentence in noise task, as did Petersen and colleagues during a digits in noise memory task and Wöstmann et al. (2016) during a digits in single talker distraction. The alpha ERS sources described here are therefore consistent with these studies. The alpha ERS has previously been ascribed to play a role as a “suppressor” of irrelevant information in sensory stimulus processing (e.g., modality switching; Mazaheri et al., 2014) or in memory (Bonnefond and Jensen, 2012) tasks. In their review, Strauß et al. (2014) suggest that if speech and noise are processed as separate channels (Shinn-Cunningham, 2008), alpha ERS may be a mechanism by which the brain suppresses the noise channel while preserving the speech channel. According to this model, noise suppression may be achieved by alpha ERS during the active condition of a DiN paradigm. The finding that ERS was not observed during selective attention to the movie (i.e., the passive condition) suggests that alpha ERS may only arise or be effective during a sustained, homogeneous stimulus.

A long time constant for ERS is supported by the data recorded in this study where alpha ERS was associated with a non-spatial, object-based parietal attention network. Although auditory and visual spatial attention associated with parietal activation have been well described (Colby and Goldberg, 1999), parietal activation without a spatial feature has also been previously described (Farah et al., 1989; Shomstein and Yantis, 2006). One interpretation of the nature of the ERS and ERD in our DiN task may be related to neural activation and inhibition of “what” and “where”, dual-pathway pathways in the auditory system (Rauschecker and Tian, 2000). ERD and ERS sources reported here are similar to regions identified in a meta-analysis study of PET and fMRI studies that examined the auditory dual-pathway of spatial and non-spatial auditory object processing (Arnott et al., 2004). From this perspective, the non-spatial, object identification (i.e., digits in the DiN) should be enhanced for optimal performance while the distracting or irrelevant spatial processing should be suppressed for optimal performance on DiN. Alpha ERD and ERS may provide a mechanism for this dorsal enhancement and ventral suppression (e.g., Jensen and Mazaheri, 2010).

### Correct vs. Incorrect Digit Identification

In an effort to understand what relative roles ERS and ERD play on comprehension of SiN, we separated correct and incorrect trials and found that alpha ERD in temporal areas consistently increased in magnitude (greater ERD) on correct trials relative to incorrect trials. This result indicates that gain mechanisms in left temporal lobe are related to comprehension. Such a relationship is also supported by the significant correlation between alpha ERD and DiN performance. Similar temporal alpha ERD relationships with overall speech comprehension during listening to noise vocoded stimuli have been previously reported (Obleser and Weisz, 2012; Becker et al., 2013). Wöstmann et al. (2016) found that the degree of alpha lateralization (relative activation across left and right sensors) was correlated with performance on a dichotic digits identification task. These data are consistent with our finding that temporal alpha is related to speech stimulus identification.

## Conclusion

The results of this study demonstrate: (1) that selective auditory attention can be indexed using brain oscillatory power changes during active relative to passive attention. To our knowledge, such passive/active differences have not previously been reported; (2) Analyzing oscillatory activity based on brain source rather than sensor level modeling can reveal robust relationships with behavior. Specifically, alpha ERD and its relationship to DiN intelligibility became apparent only when we examined brain sources; and (3) A better understanding of the neural dynamics related to SiN perception in NH populations is a useful step in understanding clinical populations with SiN deficits. This study is the first to relate neural processes with performance on the DiN, a hearing test that is becoming increasingly common.

## Author Contributions

AD, DSK and DRM: experimental design, data analysis, manuscript preparation; MLS: experimental design, data analysis, manuscript preparation, performed the experiment.

## Funding

Funding for this work was provided by National Institute on Deafness and Other Communication Disorders (NIDCD) grant R01 1R01DC014078 to DRM and the Hearing Health Foundation to AD.

## Conflict of Interest Statement

The authors declare that the research was conducted in the absence of any commercial or financial relationships that could be construed as a potential conflict of interest.
